# Time trends in socio-economic inequalities in stunting prevalence: analyses of repeated national surveys

**DOI:** 10.1017/S1368980014002924

**Published:** 2014-12-18

**Authors:** María Clara Restrepo-Méndez, Aluísio JD Barros, Robert E Black, Cesar G Victora

**Affiliations:** 1 International Center for Equity in Health, Federal University of Pelotas, Rua Marechal Deodoro 1160, 3° Piso, 96020–220 Pelotas, Brazil; 2 Institute for International Programs, Bloomberg School of Public Health, Johns Hopkins University, Baltimore, MD, USA

**Keywords:** Stunting, Malnutrition, Nutritional status, Child, Socio-economic factors

## Abstract

**Objective:**

Much is known about national trends in child undernutrition, but there is little information on how socio-economic inequalities are evolving over time. We aimed to assess socio-economic inequalities in stunting prevalence over time.

**Design:**

We selected nationally representative surveys carried out since the mid-1990s for which information was available on asset indices and on child anthropometry. We identified twenty-five countries that had at least two surveys over an interval of 10 years or more, totalling eighty-seven surveys. Stunting prevalence was calculated according to wealth quintiles. Absolute and relative inequalities were calculated and time trends were obtained by regression.

**Setting:**

Nationally representative household surveys from twenty-five low- and middle-income countries.

**Subjects:**

Children <5 years of age.

**Results:**

National prevalence declined significantly in twenty-two of the twenty-five countries. In eighteen out of twenty-five countries, relative reductions were higher among the rich than among the poor. Overall, there was no indication that inequalities improved. Striking examples are Nepal, with a 17·0 percentage points decline in stunting per decade, but where inequalities increased sharply; and Brazil, where stunting fell by 6·7 percentage points and inequalities were all but eliminated.

**Conclusions:**

Global progress in reducing stunting has not been accompanied by improved equity, but countries varied markedly in how successful they were in reducing prevalence among the poorest children. It is important to document how some countries were able to reduce inequalities, so that these lessons can be used to foster global progress, particularly in light of the increased importance of within-country inequalities in the post-2015 agenda.

The effort towards the Millennium Development Goals has been a key driver of initiatives for the production of timely information on health indicators. Nevertheless, the Millennium Development Goals have been criticized for focusing on national-level targets, while failing to emphasize within-country inequalities^(^ ^1^ ^,^ ^2^ ^)^. Regional and national averages can hide important inequalities among sub-national population groups (e.g. by household wealth and area of residence)^(^ ^3^ ^)^. Therefore there is an increasing interest in understanding patterns and trends in inequalities in health indicators and in using these insights to guide programmes to target, deliver and monitor health in the most vulnerable groups^(^ ^3^ ^,^ ^4^ ^)^. Equity considerations have been highly prominent in the ongoing discussion process that will shape the next round of global goals for the post-2015 period – the Sustainable Development Goals (www.worldwewant2015.org).

Nearly half of all deaths among children under the age of 5 years are attributable to undernutrition. Globally, 26 % of children <5 years of age were stunted in 2011^(^ ^4^ ^)^. But this burden is not evenly distributed around the world. Three-quarters of the world’s stunted children live in sub-Saharan Africa and South Asia^(^ ^4^ ^)^. Stunting reflects linear growth deficits, and is increasingly being recognized as the most important anthropometric indicator for child nutrition^(^ ^5^ ^)^ and the most sensitive indicator of the quality of a child’s life^(^ ^4^ ^)^. The three proximate determinants of child growth – food, illness and care – are strongly related to social and economic conditions, and as a consequence show marked variability among different social groups within virtually every low- and middle-income country^(^ ^5^ ^)^.

Increased attention to monitoring and accountability regarding child health and nutrition in low- and middle-income countries has led to a marked increase in the number and frequency of national surveys, which are now available for about 100 different countries. Most surveys include information that allows classification of households according to socio-economic position and therefore the investigation of social inequalities in health and nutrition^(^ ^3^ ^,^ ^6^ ^)^.

The increased availability of population-based data, as discussed, allows investigation of levels and trends in socio-economic inequalities in child stunting at a scale that was never possible in the past. A recent publication addressed urban/rural disparities in stunting and underweight in low- and middle-income countries^(^ ^7^ ^)^. Here, we report on wealth-based inequalities in stunting prevalence for twenty-five countries with data available from the mid-1990s to the present day, with special attention to how absolute and relative disparities evolve over time.

## Methods

### Data sources

We used data from the Demographic and Health Surveys (DHS; http://www.measuredhs.com/aboutsurveys/dhs/start.cfm) and Multiple Indicator Cluster Surveys (MICS; http://www.childinfo.org/), both of which are cross-sectional, nationally representative household surveys with information on maternal and child health. These surveys have been conducted about every 3 to 5 years since the mid-1980s and mid-1990s, respectively. Data were obtained in these surveys through standardized interviews with women aged 15–49 years. Height and weight measurements were collected for children through standardized methods. Recumbent length was recorded for children <2 years of age or shorter than 85 cm. Standing height was measured for all other children^(^ ^6^ ^,^ ^8^ ^)^.

We selected countries for which at least two surveys were available, with at least 10 years between the earlier and the most recent survey since the mid-1990s, and for which information was available on asset indices and on child anthropometry. Twenty-five countries had such information. If more than two surveys for the same country were available then all were included in the analyses (see online supplementary material, Supplemental Table 1); for example, for Malawi we included three DHS surveys (2000, 2004 and 2010) and one MICS survey (2006). In four countries (Benin, Cameroon, Madagascar and Mali), anthropometric data were restricted to children <3 years of age in the earliest survey and the same age range was analysed in subsequent surveys. In the remaining twenty-two countries, all children <5 years of age were the target group. Countries were classified into five regions according to the UNICEF groupings (Eastern and Southern Africa, West and Central Africa, Middle East, South and East Asia, and Latin America and Caribbean; www.unicef.org).

### Dependent variable

Children were classified as stunted if their height-for-age was more than 2 sd below the median height for age and sex as would be expected based on the WHO Child Growth Standards (www.who.int/childgrowth); that is, if height-for-age *Z*-score (HAZ)<−2.

### Independent variable

As a measure of socio-economic position, we used a wealth index which is the household asset-based wealth scores as calculated by the original DHS or MICS survey team. These scores are based on country-specific sets of household assets and generated by principal component analysis^(^ ^9^ ^)^. Each household is then assigned an asset score and samples are broken down into quintiles based on this asset score. We refer to the first quintile (Q1) as the poorest quintile/poorest 20 % and the fifth quintile (Q5) as the wealthiest quintile/wealthiest 20 %.

### Statistical analysis

#### Stunting prevalence

We calculated the stunting prevalence for each country as a whole and stratified by quintiles of wealth index for each year.

#### Measures of inequalities

Two indicators of inequality were estimated: (i) an indicator of absolute inequality, the slope index of inequality (SII); and (ii) an indicator of relative inequality, the concentration index (CIX)^(^ ^10^ ^)^.

Both SII and CIX take into account all socio-economic groups (e.g. quintiles of wealth index) rather than only the extreme groups (e.g. Q1 and Q5), as is the case for simple measures of inequalities such as the absolute inequality (which is the arithmetic difference between the top and bottom wealth quintiles) and the relative inequality (which is the rate ratio between the top and bottom wealth quintiles)^(^ ^10^ ^)^.

The SII is typically derived through performing the linear regression of the health outcome *v*. the midpoints of the ranks obtained by ordering the sample by the explanatory variable (e.g. quintiles of wealth index) when using grouped data. Because stunting is a proportion, we estimated the SII using logistic regression to avoid predicting implausible values below zero or above one^(^ ^11^ ^)^. The SII estimates the absolute difference in stunting prevalence, expressed as percentage points, between individuals at the top and bottom of the wealth scale. For example, an SII of −20 indicates that prevalence among the wealthiest children is 20 percentage points lower than among the poorest ones.

The CIX was calculated in its relative formulation, with no corrections^(^ ^11^ ^)^. The CIX uses an analogous approach to the Gini index, by ranking individuals according to socio-economic position on the *x*-axis and for example cumulative health condition on the *y*-axis. Thus, for example, if every wealth quintile had 20 % of all the prevalence of a health condition distributed in a population, the line would be exactly on the diagonal and there would be no inequality^(^ ^11^ ^)^. This index is expressed on a scale ranging from −100 to 100; a value of 0 represents perfect equality, whereas negative values indicate that poor individuals have greater prevalence than rich individuals^(^ ^12^ ^)^.

#### Trends in stunting prevalence, and absolute and relative inequalities

To assess trends in stunting prevalence over time, we estimated the annual change by performing the regression of the observed values in each survey *v*. the year of the survey. This is because three or more surveys were available for most countries (see online supplementary material, Supplemental Table 1). Then we expressed the regression slope as the absolute change in percentage points over a 10-year interval. In addition, we estimated the annual change for the poorest 20 % (Q1) and for the wealthiest 20 % (Q5).

We also compared trends in absolute (SII) *v*. relative (CIX) inequality. Annual changes in SII and CIX were derived from linear regression, including one data point per available survey, similar to our estimation of trends in stunting prevalence. For absolute and relative changes the regression slope was expressed as the percentage change over a 10-year interval after the earliest survey.

The statistical software package Stata version 12·1 was used to perform all calculations, for which we took into account the survey design, including sampling weights and clustering. All point estimates of prevalence and inequality indices were calculated with standard errors from the original data sets. Significance of changes over time was calculated through *t* tests based on the means and standard errors for the earliest and latest available surveys. Spearman correlation coefficients were used for assessing the association among rates of decline in different indicators.

All analyses are based on publicly available data from national surveys. Ethical clearance was the responsibility of the institutions that administered the surveys.

## Results

Twenty-five countries with eighty-seven surveys were included in the analyses. Supplemental Table 1 (see online supplementary material) displays the survey year, type (DHS or MICS), sample size and age range of the children. Two countries had two surveys, eleven had three, ten had four and two countries had five surveys.

**Fig. 1:**
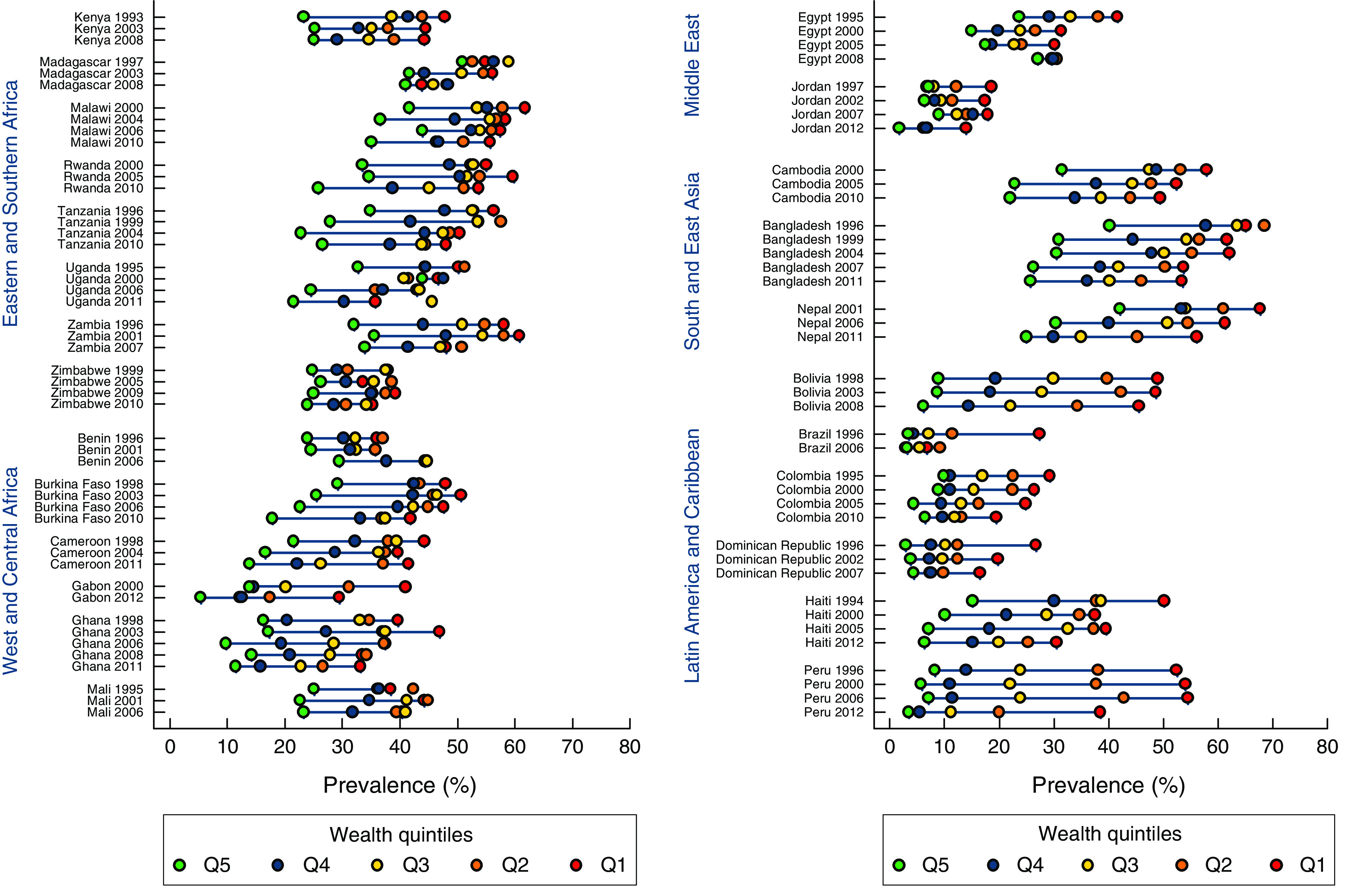
Changes over time in the prevalence of stunting among children <5 years of age by wealth quintile. Data are from nationally representative household surveys in twenty-five low- and middle-income countries where least two surveys were available with at least 10 years between the earlier and the most recent survey since the mid-1990s, and for which information was available on asset indices and on child anthropometry. The coloured dots show the average prevalence in each wealth quintile; Q1 is the poorest quintile/poorest 20 % and Q5 is the wealthiest quintile/wealthiest 20 %. The horizontal lines connect the wealthiest and poorest quintiles; the longer the line between the two groups, the greater the absolute inequality. Note: Peru 2006, this is the mid-point in time of the continuous Demographic and Health Survey from 2004 to 2008; results are based on the whole period from 2004 to 2008

Figure 1 presents five-dot charts of trends in stunting prevalence by wealth quintile in all twenty-five countries. In most surveys, stunting increases monotonically with decreasing wealth. In all but two surveys (Brazil 2006 and Jordan 1997 – both of which have low stunting prevalence and little inequality), the lowest prevalence is observed in the wealthiest quintile. In sixty-four of the eighty-seven surveys, the poorest quintile showed the highest prevalence. The width of the bars represents absolute inequality, which is very marked in countries such as Peru and Bolivia and relatively small in the Brazil 2006 and Egypt 2008 surveys, for example.

Time trends from Fig. 1 are summarized numerically in Table 1 and in greater detail in the online supplementary material, Supplemental Table 2. Negative values for changes in stunting prevalence indicate improved nutritional status. Countrywide stunting prevalence declined in twenty-two countries. The decline was particularly marked in Nepal where stunting fell by 17 percentage points in a 10-year period. Stunting prevalence declined both among the poorest and the wealthiest in the vast majority of countries. In three countries prevalence increased, of which in one the increase was significant (Benin).

**Table 1:** Changes over a 10-year interval in the prevalence of stunting among children <5 years of age, and absolute and relative inequalities, by country

		Change in stunting prevalence									
				Absolute change		Relative change		Change in wealth-related summary measures			
Region	Country	Overall	*P* value*	Q1 (poorest)	Q5 (wealthiest)	Q1 (poorest)	Q5 (wealthiest)	SII	*P* value*	CIX	*P* value*
East and Southern Africa	Kenya	−3·2	0·004	−2·5	1·4	−5·3	5·9	−0·2	0·87	−1·1	0·4
	Madagascar	−6·3	<0·001	−8·1	−8·4	−14·8	−16·5	−2·2	0·81	−0·8	0·77
	Malawi	−6·8	<0·001	−6·0	−5·0	−9·8	−11·9	0·8	0·87	−0·9	0·34
	Rwanda	−4·1	0·002	−1·3	−7·6	−2·4	−22·8	−9·2	0·02	−4·6	<0·001
	Tanzania	−5·7	<0·001	−5·7	−6·8	−10·1	−19·6	2·3	0·69	−0·3	0·60
	Uganda	−7·9	<0·001	−8·7	−10·1	−17·2	−30·9	1·2	0·14	−1·0	0·83
	Zambia	−3·2	0·03	−9·5	1·5	−16·4	4·8	14·2	<0·001	4·5	0·003
	Zimbabwe	0·3	0·41	−0·2	−0·8	−0·5	−3·2	0·6	0·54	0·2	0·85
West and Central Africa	Benin	10·2	<0·001	13·3	5·2	37·0	21·6	−9·2	0·05	−1·8	0·44
	Burkina Faso	−5·9	<0·001	−5·1	−9·4	−10·5	−32·0	−4·2	0·32	−3·4	0·04
	Cameroon	−3·8	0·008	1·3	−6·4	3·0	−29·7	−11·2	0·007	−7·8	<0·001
	Gabon	−7·5	<0·001	−9·5	−7·1	−23·2	−51·1	7·0	0·05	−4·4	0·27
	Ghana	−7·4	<0·001	−7·8	−4·1	−19·7	−25·1	3·5	0·60	−2·9	0·21
	Mali	1·8	0·11	5·3	−1·3	13·7	−5·3	−6·6	0·06	−1·6	0·36
Middle East	Egypt	−3·3	<0·001	−8·1	−2·1	−19·6	9·0	13·7	<0·001	6·1	<0·001
	Jordan	−1·8	<0·001	−2·4	−3·7	−12·9	52·4	2·7	0·47	3·4	0·59
South Asia and East Asia	Bangladesh	−11·8	<0·001	−7·9	−8·6	−12·1	−21·4	−2·1	0·16	−3·3	<0·001
	Nepal	−17·0	<0·001	−11·5	−17·2	−17·0	−40·8	−10·7	0·02	−8·6	<0·001
	Cambodia	−10·5	<0·001	−8·5	−9·6	−14·7	−30·5	−6·7	0·15	−5·0	0·007
Latin America and Caribbean	Bolivia	−5·7	<0·001	−3·5	−2·7	−7·2	−30·2	−2·0	0·53	−5·8	<0·001
	Brazil	−6·7	<0·001	−20·7	−0·2	−75·4	−7·1	33·0	<0·001	36·9	<0·001
	Colombia	−4·5	<0·001	−6·2	−2·9	−21·3	−29·3	6·7	<0·001	2·4	0·06
	Dominican Republic	−3·3	<0·001	−9·5	1·3	−35·4	45·7	13·9	<0·001	10·5	0·003
	Haiti	−8·0	<0·001	−9·8	−4·9	−19·5	−32·3	2·1	0·2	−3·2	0·05
	Peru	−8·7	<0·001	−9·0	−2·6	−17·2	−31·6	6·1	0·003	−6·6	<0·001

SII, slope index of inequality (for absolute inequality); CIX, concentration index (for relative inequality). Data are from nationally representative household surveys in twenty-five low- and middle-income countries where least two surveys were available with at least 10 years between the earlier and the most recent survey since the mid-1990s, and for which information was available on asset indices and on child anthropometry.

* Student’s *t* test comparing the earliest and latest survey in the country.

Absolute and relative summary measures of inequality at any given point in time are usually negative, because stunting is more common among the poor than the rich. However, reductions in inequality result in positive values for annual change; that is, the most recent surveys show values that are still negative but that are closer to zero than the earlier surveys.

In eighteen out of twenty-five countries, relative reductions, but not absolute reductions, were higher in the wealthiest 20 % (Q5) than in the poorest 20 % (Q1). In terms of SII, fourteen countries showed improvement in absolute inequalities, of which seven were significant (Zambia, Gabon, Egypt, Brazil, Colombia, Dominican Republic and Peru). The remaining eleven countries showed increased absolute inequalities, of which four were significant (Rwanda, Benin, Cameroon and Nepal). The most marked improvement was in Brazil, whereas the sharpest deterioration in equality was in Nepal and Cameroon.

In terms of relative inequalities (CIX), only seven countries showed improvement, which was significant in four (Zambia, Egypt, Brazil and Dominican Republic). The remaining eighteen countries showed increased inequalities, for nine of which there were significant differences (Rwanda, Burkina Faso, Cameroon, Bangladesh, Nepal, Cambodia, Bolivia, Haiti and Peru).

Changes in prevalence were not strongly correlated with changes in absolute inequality (Spearman correlation coefficient *ρ*=−0·04; *P*=0·7) and moderately correlated with changes in relative inequality (*ρ*=0·6; *P*=<0·001; see online supplementary material, Supplemental Table 3). For example, Bangladesh, Brazil, Gabon, Haiti and Uganda had similar annual reductions in stunting but their performance in terms of inequalities varied widely (Fig. 1 and Table 1).

**Fig. 2:**
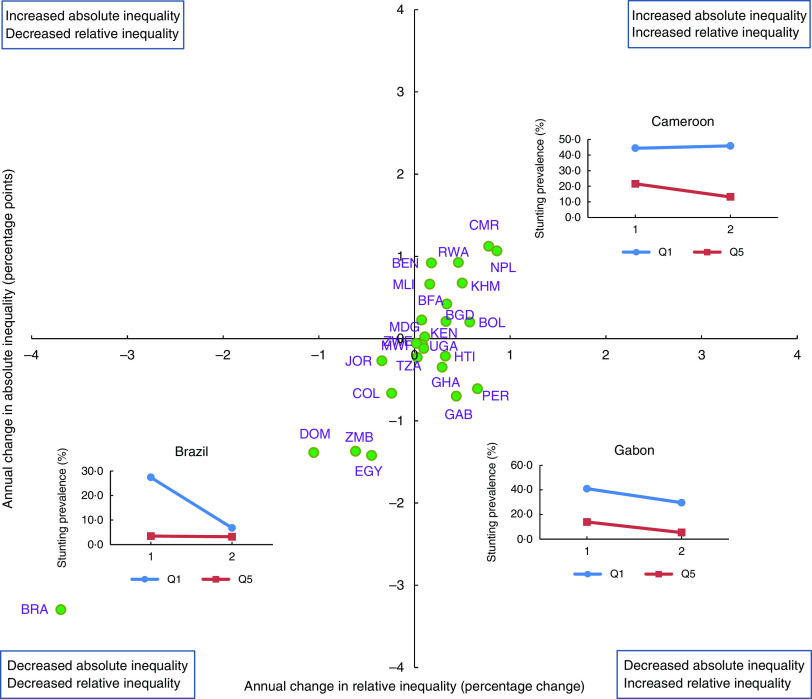
Scatter plot of changes in absolute (SII) and relative (CIX) inequality in the prevalence of stunting among children <5 years of age by country. Data are from nationally representative household surveys in twenty-five low- and middle-income countries where least two surveys were available with at least 10 years between the earlier and the most recent survey since the mid-1990s, and for which information was available on asset indices and on child anthropometry. Annual changes in the slope index of inequality (SII) for absolute inequality and the concentration index (CIX) relative inequality were derived from linear regression, including one data point per available survey, with the regression slope expressed as the percentage change over a 10-year interval after the earliest survey. The inserts show examples of countries where there was an improvement in absolute and relative terms (Brazil), where both measures worsened (Cameroon) and where absolute inequality remained stable but relative inequalities declined (Gabon); Q1 is the poorest quintile/poorest 20 % and Q5 is the wealthiest quintile/wealthiest 20 %. See online supplementary material, Supplemental Table 3 for the three-letter country codes

Figure 2 shows a scatter diagram of changes in the two dimensions of inequalities (see online supplementary material, Supplemental Table 2 for more details). Because the CIX assumes negative value in the case of ill health (due to poorer groups being affected more than wealthier groups), we multiplied the estimates shown in the diagram by −1 to facilitate interpretation. Equity improved significantly according to both indicators in Zambia, Egypt, Brazil and Dominican Republic. The worst performers included Rwanda, Cameroon and Nepal, where both indicators showed significant increases in inequality. The inserts in Fig. 2 show examples of countries where there was an improvement in absolute and relative terms (Brazil), where both measures worsened (Cameroon) and where absolute inequality remained stable but relative inequalities declined (Gabon). Countries that showed most improvement were those from Latin America.

## Discussion

The present systematic analysis of countries with two surveys spaced by at least 10 years, in which measures of stunting prevalence and socio-economic inequality were available, builds upon earlier analyses presented in the *Lancet* Nutrition Series 2013^(^ ^5^ ^)^. The choice of a 10-year or longer interval between surveys was aimed at maximizing the likelihood of documenting changes in inequality patterns, assuming that these patterns are unlikely to change in the short term given the long duration of the process of linear growth retardation^(^ ^13^ ^,^ ^14^ ^)^.

In agreement with previous analyses, stunting prevalence was found to be declining in most countries^(^ ^5^ ^,^ ^15^ ^)^. The conclusions regarding inequalities, however, are less encouraging. We found little association between the rate of decline in stunting and improvement in equity. This is in contrast with a recent analysis showing that countries which managed to rapidly increase population coverage with essential interventions directed at pregnant women and their children, did so largely by reducing within-country inequalities^(^ ^12^ ^)^.

When interpreting changes in inequality patterns, it is essential to assess absolute and relative changes, because these may evolve in different directions and are open to different interpretations^(^ ^10^ ^)^. For example, in a country where stunting prevalence declined from say 50 % to 30 % in the poorest quintile, and from 10 % to 5 % in the wealthiest quintile, the absolute change would be larger for the poor (20 percentage points) than for the rich (5 percentage points). However, the relative reduction in the poor would be of 40 % (from 50 % to 30 %) and that for the rich of 50 % (from 10 % to 5 %). One may either celebrate the fact that the absolute drop in percentage points was much greater in the poor than in the rich, or regret the observation that the relative gap has increased from fivefold to sixfold. This is especially noticeable when using simple measures of inequalities that take into account only the top and bottom extremes of the socio-economic distribution of the population under study. In the present study we report annual changes on inequalities using SII and CIX indices, which take into account not only the extremes groups of the socio-economic distribution (e.g. Q1 and Q5) but also the intermediate population groups (e.g. Q2 to Q4). By using information on the whole population, these indices are less sensitive to changes in the number of individuals in each stratification category and also less likely to be affected when a prevalence of the wealthiest group is close to zero. The latter case is especially important for relative inequalities.

When prevalence declines over time, a common finding is that absolute inequalities between poor and rich tend to fall faster than relative inequalities. The best possible combination is when both types of measures of inequality show improvement. This was noticeably the case for Brazil, which was a true and positive outlier in the present analyses. Earlier analyses suggest that several factors played a role in explaining why there was a marked overall decline in stunting prevalence, mostly due to a decline in the poorest groups of Brazilian society^(^ ^16^ ^)^. The factors that are deemed responsible for such progress include general socio-economic progress, improvements in female education, lower fertility, urbanization and interventions in the health and other sectors including conditional cash transfers and universal health-care coverage^(^ ^16^ ^,^ ^17^ ^)^. To a lesser extent, improvements in both relative and absolute inequalities, against a backdrop of falling stunting prevalence, was also observed in the Dominican Republic.

Egypt and Zambia showed improvements in both relative and absolute inequality. In both countries stunting prevalence tended to decrease during the time period based on the regression analyses of data from four surveys in Egypt and three in Zambia. However, in both countries (Fig. 1) there was some evidence that stunting in the wealthiest quintile increased recently whereas the poor improved or remained stable, and as a consequence inequality was reduced. In Egypt the reason for the increase in stunting prevalence requires further research; however, a factor that may in part be responsible for the increase among most of the socio-economic groups was the abrupt disruption in the supplies of poultry and eggs which followed the culling of millions of chickens and other poultry in response to the avian influenza outbreak experienced in 2006^(^ ^18^ ^)^.

Among the worst performers in terms of equity, Bangladesh and in particular Nepal showed rapid declines in overall stunting prevalence, but this was largely due to rapid relative improvement among the richest (Table 1 and Fig. 1) which was not as rapid among the poor. Likewise, in Rwanda prevalence fell moderately (4·1 percentage points in a 10-year period) but the poor were left behind with a decline of only 1·3 percentage points). In Benin (Fig. 1) there was a marked deterioration in nutritional status among the poorest, which was likely due to famine in rural parts of the country in the year preceding the 2006 survey^(^ ^19^ ^)^, whereas the richest were little affected in terms of stunting prevalence.

A recent publication used a Bayesian hierarchical mixture model to describe time trends in height- and weight-for-age *Z*-scores among children <5 years of age from low- and middle-income countries^(^ ^7^ ^)^. The authors resorted to a complex modelling procedure to impute data for countries and/or time periods without available surveys. Their overall conclusion is that absolute urban/rural inequalities – expressed as *Z*-score differences between urban and rural residents – were reduced in Latin America and South Asia, but not in other regions of the world. One may expect urban/rural differentials to evolve in tandem with wealth inequities. Our findings on the latter showed improvement in Latin America, both in terms of relative and absolute inequalities. We did not find a consistent narrowing of inequalities in the three South and East Asian countries included in the analyses (Bangladesh, Nepal and Cambodia), nor in Africa. This discrepancy in conclusions between the two sets of analyses is likely due to differences in the statistical approach carried out. We relied on actual data from surveys, rather than the complex modelling approach favoured by the authors of the Bayesian modelling exercise in which prevalence was imputed for some countries and years^(^ ^7^ ^)^.

In most countries we observed that the time trends in stunting prevalence (SII and CIX) were relatively linear. However, for Madagascar and Uganda time trends in SII and CIX were visibly non-linear (see online supplementary material, Supplemental Table 2). A potential false assumption of linearity may have biased our findings. However, we believe that trying to fit polynomials to the countries – when linear trends are present in virtually all of them – is not warranted, since we would not get a mean annual change that is comparable across countries.

The wide variability in changes in equity, among countries with similar rates of reduction in stunting in the overall population, deserves further studies about which interventions or policy changes (in the health/nutrition or in other sectors) may have led to success. Monitoring of trends disaggregated by wealth, urban/rural residence and other relevant stratifiers can make an important contribution to assessment of trends at national level. This is particularly relevant in light of the greater focus on equity which will likely be a key aspect of the post-2015 agenda^(^ ^20^ ^,^ ^21^ ^)^.

## Supplementary material

For supplementary material accompanying this paper visit https://doi.org/10.1017/S1368980014002924. click here to view supplementary material
