# Supplementary material for: Time trends in socio-economic inequalities in stunting prevalence: analyses of repeated national surveys
Source: Public Health Nutr. 2014 Dec 18;18(12):2097–104. doi: 10.1017/S1368980014002924 (PMC4909139; doi:10.1017/S1368980014002924)
Supplement: Supplementary file 1 [file S1368980014002924sup001.doc]

**Supplemental T**able 1. Stunting prevalence by country, survey year and type of survey

| **Region** | **Country** | **Study period^†^** | **Child age** | **Year** | **Source** | **N** | **Year** | **Source** | **N** | **Year** | **Source** | **N** | **Year** | **Source** | **N** | **Year** | **Source** | **N** |
| --- | --- | --- | --- | --- | --- | --- | --- | --- | --- | --- | --- | --- | --- | --- | --- | --- | --- | --- |
| Eastern and Southern Africa | Kenya | 15 | 5 | 1993 | DHS | 4757 | 2003 | DHS | 4893 | 2008 | DHS | 4958 |  |  |  |  |  |  |
|  | Madagascar | 11 | 3 | 1997 | DHS | 3074 | 2003 | DHS | 3052 | 2008 | DHS | 3965 |  |  |  |  |  |  |
|  | Malawi | 10 | 5 | 2000 | DHS | 9343 | 2004 | DHS | 7873 | 2006 | MICS | 21426 | 2010 | DHS | 4559 |  |  |  |
|  | Rwanda | 10 | 5 | 2000 | DHS | 6249 | 2005 | DHS | 3672 | 2010 | DHS | 4117 |  |  |  |  |  |  |
|  | Tanzania | 14 | 5 | 1996 | DHS | 5324 | 1999 | DHS | 2578 | 2004 | DHS | 7368 | 2010 | DHS | 6895 |  |  |  |
|  | Uganda | 16 | 5 | 1995 | DHS | 4785 | 2000 | DHS | 5610 | 2006 | DHS | 2397 | 2011 | DHS | 2069 |  |  |  |
|  | Zambia | 11 | 5 | 1996 | DHS | 5455 | 2001 | DHS | 5205 | 2007 | DHS | 5158 |  |  |  |  |  |  |
|  | Zimbabwe | 11 | 5 | 1999 | DHS | 2538 | 2005 | DHS | 3905 | 2009 | MICS | 6173 | 2010 | DHS | 4275 |  |  |  |
| West and Central Africa | Benin | 10 | 3 | 1996 | DHS | 2294 | 2001 | DHS | 2603 | 2006 | DHS | 10052 |  |  |  |  |  |  |
|  | Burkina Faso | 12 | 5 | 1998 | DHS | 3787 | 2003 | DHS | 8236 | 2006 | MICS | 4253 | 2010 | DHS | 6660 |  |  |  |
|  | Cameroon | 13 | 3 | 1998 | DHS | 1935 | 2004 | DHS | 2035 | 2011 | DHS | 4180 |  |  |  |  |  |  |
|  | Gabon | 12 | 5 | 2000 | DHS | 3028 | 2012 | DHS | 2792 |  |  |  |  |  |  |  |  |  |
|  | Ghana | 13 | 5 | 1998 | DHS | 2576 | 2003 | DHS | 2923 | 2006 | MICS | 3200 | 2008 | DHS | 2326 | 2011 | MICS | 7338 |
|  | Mali | 11 | 3 | 1995 | DHS | 4664 | 2001 | DHS | 5974 | 2006 | DHS | 8926 |  |  |  |  |  |  |
| Middle East | Egypt | 13 | 5 | 1995 | DHS | 9748 | 2000 | DHS | 10231 | 2005 | DHS | 12225 | 2008 | DHS | 9207 |  |  |  |
|  | Jordan | 15 | 5 | 1997 | DHS | 5512 | 2002 | DHS | 4636 | 2007 | DHS | 4077 | 2012 | DHS | 5943 |  |  |  |
| South Asia and East Asia | Bangladesh | 15 | 5 | 1996 | DHS | 4760 | 1999 | DHS | 5436 | 2004 | DHS | 6000 | 2007 | DHS | 5242 | 2011 | DHS | 7683 |
|  | Nepal | 11 | 5 | 2001 | DHS | 6238 | 2006 | DHS | 5024 | 2011 | DHS | 2380 |  |  |  |  |  |  |
|  | Cambodia | 10 | 5 | 2000 | DHS | 3263 | 2005 | DHS | 3347 | 2010 | DHS | 3718 |  |  |  |  |  |  |
| Latin America and Caribbean | Bolivia | 10 | 5 | 1998 | DHS | 5776 | 2003 | DHS | 9058 | 2008 | DHS | 7815 |  |  |  |  |  |  |
|  | Brazil | 10 | 5 | 1996 | DHS | 3831 | 2006 | DHS | 10404 |  |  |  |  |  |  |  |  |  |
|  | Colombia | 15 | 5 | 1995 | DHS | 4420 | 2000 | DHS | 4076 | 2005 | DHS | 11575 | 2010 | DHS | 14229 |  |  |  |
|  | Dominican Rep | 11 | 5 | 1996 | DHS | 3476 | 2002 | DHS | 8600 | 2007 | DHS | 8761 |  |  |  |  |  |  |
|  | Haiti | 18 | 5 | 1994 | DHS | 2797 | 2000 | DHS | 5332 | 2005 | DHS | 2401 | 2012 | DHS | 3753 |  |  |  |
|  | Peru | 16 | 5 | 1996 | DHS | 13434 | 2000 | DHS | 10465 | 2006* | DHS | 5108 | 2012 | DHS | 8417 |  |  |  |
| †Period (in years) from first to last survey | | | | |  |  |  |  |  |  |  |  |  |  |  |  |  |  |
| *Peru 2006: This the mid-point in time of the continuous DHS from 2004 to 2008. Results are based on the whole period from 2004-2008. | | | | | | | | | | | | | | | | | | |

**Supplemental T**able 2. Changes in stunting prevalence, SII and CIX over time

| **Region** | **Country** |  | | | | | | **Annual change** |
| --- | --- | --- | --- | --- | --- | --- | --- | --- |
| Eastern and Southern Africa |  | **Year** | **1993** | **2003** | **2008** |  |  |  |
|  | Kenya | Stunting (%) | 39·9 | 36·0 | 35·3 |  |  | -0·3 |
|  |  | SII | -23·4 | -21·9 | -24·1 |  |  | 0·0 |
|  |  | CIX | -10·1 | -10·7 | -11·9 |  |  | -0·1 |
|  |  | **Year** | **1997** | **2003** | **2008** |  |  |  |
|  | Madagascar | Stunting (%) | 55·0 | 50·4 | 48·1 |  |  | -0·6 |
|  |  | SII | -0·5 | -18·9 | -1·9 |  |  | -0·2 |
|  |  | CIX | -0·3 | -5·9 | -0·8 |  |  | -0·1 |
|  |  | **Year** | **2000** | **2004** | **2006** | **2010** |  |  |
|  | Malawi | Stunting (%) | 54·3 | 52·2 | 53·0 | 47·1 |  | -0·7 |
|  |  | SII | -21·1 | -22·8 | -14·9 | -21·8 |  | 0·1 |
|  |  | CIX | -6·5 | -7·4 | -4·9 | -8·0 |  | -0·1 |
|  |  | **Year** | **2000** | **2005** | **2010** |  |  |  |
|  | Rwanda | Stunting (%) | 48·1 | 50·8 | 44·0 |  |  | -0·4 |
|  |  | SII | -22·4 | -24·6 | -31·7 |  |  | -0·9 |
|  |  | CIX | -8·0 | -8·2 | -12·6 |  |  | -0·5 |
|  |  | **Year** | **1996** | **1999** | **2004** | **2010** |  |  |
|  | Tanzania | Stunting (%) | 49·5 | 48·2 | 44.1 | 41·6 |  | -0·6 |
|  |  | SII | -22·7 | -29·7 | -25.1 | -20·9 |  | 0·2 |
|  |  | CIX | -7·7 | -11·1 | -9.4 | -8·5 |  | -0·03 |
|  |  | **Year** | **1995** | **2000** | **2006** | **2011** |  |  |
|  | Uganda | Stunting (%) | 45·0 | 44·4 | 37·3 | 33·2 |  | -0·8 |
|  |  | SII | -20·1 | -0·6 | -15·9 | -12·2 |  | 0·1 |
|  |  | CIX | -7·7 | -0·5 | -6·7 | -7·2 |  | -0·1 |
|  |  | **Year** | **1996** | **2001** | **2007** |  |  |  |
|  | Zambia | Stunting (%) | 48·5 | 52·6 | 45·3 |  |  | -0·3 |
|  |  | SII | -31·3 | -27·3 | -15·9 |  |  | 1·4 |
|  |  | CIX | -10·8 | -8·8 | -5·9 |  |  | 0·4 |
|  |  | **Year** | **1999** | **2005** | **2009** | **2010** |  |  |
|  | Zimbabwe | Stunting (%) | 32·3 | 33·4 | 35.0 | 31·1 |  | 0·03 |
|  |  | SII | -14·0 | -9·0 | -14.3 | -11·1 |  | 0·06 |
|  |  | CIX | -6·7 | -4·3 | -6.6 | -6·2 |  | -0·02 |
| West and Central Africa |  | **Year** | **1996** | **2001** | **2006** |  |  |  |
|  | Benin | Stunting (%) | 32·5 | 32·3 | 42·6 |  |  | 1·0 |
|  |  | SII | -14·1 | -12·3 | -23·3 |  |  | -0·9 |
|  |  | CIX | -7·5 | -6·2 | -9·3 |  |  | -0·2 |
|  |  | **Year** | **1998** | **2003** | **2006** | **2010** |  |  |
|  | Burkina Faso | Stunting (%) | 41·4 | 43·2 | 39·8 | 34·5 |  | -0·6 |
|  |  | SII | -18·3 | -22·8 | -26·5 | -22·4 |  | -0·4 |
|  |  | CIX | -7·3 | -9·2 | -11·3 | -11·0 |  | -0·3 |
|  |  |  |  |  |  |  |  |  |
|  |  |  |  |  |  |  |  |  |
|  | **Country** |  | | | | | | **Annual change** |
|  |  | **Year** | **1998** | **2004** | **2011** |  |  |  |
|  | Cameroon | Stunting (%) | 36·2 | 33·4 | 31·2 |  |  | -0·4 |
|  |  | SII | -25·2 | -23·9 | -39·4 |  |  | -1·1 |
|  |  | CIX | -11·1 | -12·7 | -21·1 |  |  | -0·8 |
|  |  | **Year** | **2000** | **2012** |  |  |  |  |
|  | Gabon | Stunting (%) | 25·1 | 16·0 |  |  |  | -0·8 |
|  |  | SII | -35·6 | -27·2 |  |  |  | 0·7 |
|  |  | CIX | -23·8 | -29·0 |  |  |  | -0·4 |
|  |  | **Year** | **1998** | **2003** | **2006** | **2008** | **2011** |  |
|  | Ghana | Stunting (%) | 30·6 | 35·1 | 27.9 | 27·5 | 22.7 | -0·7 |
|  |  | SII | -29·0 | -32·6 | -34.5 | -23·9 | -26.5 | 0·4 |
|  |  | CIX | -15·4 | -15·7 | -21.0 | -13·9 | -19.7 | -0·3 |
|  |  | **Year** | **1995** | **2001** | **2006** |  |  |  |
|  | Mali | Stunting (%) | 36·0 | 37·9 | 38·0 |  |  | 0·2 |
|  |  | SII | -15·3 | -25·3 | -22·2 |  |  | -0·7 |
|  |  | CIX | -7·1 | -11·3 | -8·6 |  |  | -0·2 |
|  |  | **Year** | **1995** | **2000** | **2005** | **2008** |  |  |
| Middle East | Egypt | Stunting (%) | 33·7 | 23·5 | 22·9 | 28·9 |  | -0·3 |
|  |  | SII | -22·4 | -19·7 | -15·1 | -2·8 |  | 1·4 |
|  |  | CIX | -11·3 | -14·3 | -11·5 | -2·1 |  | 0·6 |
|  |  | **Year** | **1997** | **2002** | **2007** | **2012** |  |  |
|  | Jordan | Stunting (%) | 11·0 | 11·4 | 14·4 | 7.6 |  | -0·2 |
|  |  | SII | -15·1 | -13·0 | -8·0 | -12.5 |  | 0·3 |
|  |  | CIX | -23·6 | -17·4 | -9·2 | -26.6 |  | 0·3 |
| South Asia and East Asia |  | **Year** | **1996** | **1999** | **2004** | **2007** | **2011** |  |
|  | Bangladesh | Stunting (%) | 60·0 | 51·2 | 50·5 | 43·0 | 41·2 | -1·2 |
|  |  | SII | -27·1 | -34·1 | -33·5 | -32·2 | -32·2 | -0·2 |
|  |  | CIX | -7·7 | -11·3 | -11·4 | -13·1 | -13·3 | -0·3 |
|  |  | **Year** | **2001** | **2006** | **2011** |  |  |  |
|  | Nepal | Stunting (%) | 57·2 | 49·2 | 40·3 |  |  | -1·7 |
|  |  | SII | -28·0 | -35·8 | -38·7 |  |  | -1·1 |
|  |  | CIX | -8·2 | -12·5 | -16·8 |  |  | -0·9 |
|  |  | **Year** | **2000** | **2005** | **2010** |  |  |  |
|  | Cambodia | Stunting (%) | 49·6 | 42·4 | 39·1 |  |  | -1·1 |
|  |  | SII | -24·6 | -32·5 | -31·3 |  |  | -0·7 |
|  |  | CIX | -8·5 | -13·1 | -13·5 |  |  | -0·5 |
| Latin America and Caribbean |  | **Year** | **1998** | **2003** | **2008** |  |  |  |
|  | Bolivia | Stunting (%) | 32·7 | 32·3 | 27·1 |  |  | -0·6 |
|  |  | SII | -45·9 | -47·9 | -47·9 |  |  | -0·2 |
|  |  | CIX | -24·0 | -24·8 | -29·8 |  |  | -0·6 |
|  |  | **Year** | **1996** | **2006** |  |  |  |  |
|  | Brazil | Stunting (%) | 12·9 | 6·2 |  |  |  | -0·7 |
|  |  | SII | -34·9 | -1·9 |  |  |  | 3·3 |
|  |  | CIX | -42·4 | -5·4 |  |  |  | 3·7 |
|  |  |  |  |  |  |  |  |  |
|  | **Country** |  | | | | | | **Annual change** |
|  |  | **Year** | **1995** | **2000** | **2005** | **2010** |  |  |
|  | Colombia | Stunting (%) | 19·6 | 18·1 | 15·3 | 13·1 |  | -0·4 |
|  |  | SII | -26·0 | -23·4 | -23·9 | -14·7 |  | 0·7 |
|  |  | CIX | -22·1 | -22·0 | -25·8 | -16·9 |  | 0·2 |
|  |  | **Year** | **1996** | **2002** | **2007** |  |  |  |
|  | Dominican Republic | Stunting (%) | 13·5 | 11·4 | 9·8 |  |  | -0·3 |
|  |  | SII | -29·1 | -18·7 | -14·0 |  |  | 1·4 |
|  |  | CIX | -35·2 | -26·8 | -23·7 |  |  | 1·1 |
|  |  | **Year** | **1994** | **2000** | **2005** | **2012** |  |  |
|  | Haiti | Stunting (%) | 36·7 | 27·8 | 28·5 | 20·9 |  | -0·8 |
|  |  | SII | -33·6 | -31·6 | -38·6 | -27·7 |  | 0·2 |
|  |  | CIX | -15·9 | -19·5 | -23·1 | -21·4 |  | -0·3 |
|  |  | **Year** | **1996** | **2000** | **2006*** | **2012** |  |  |
|  | Peru | Stunting (%) | 31·5 | 31·0 | 28.0 | 17.8 |  | -0·9 |
|  |  | SII | -54·5 | -60·4 | -58·1 | -45.9 |  | 0·6 |
|  |  | CIX | -29·9 | -33·6 | -35·8 | -41.4 |  | -0·7 |

*Peru 2006: This the mid-point in time of the continuous DHS from 2004 to 2008. Results are based on the whole period from 2004-2008.

SII: slope index of inequality for absolute inequality; CIX: concentration index for relative inequality.

**Supplemental T**able 3. Correlations among changes in stunting prevalence, SII and CIX

|  | |  |  | **Change in 10 years** | | | |
| --- | --- | --- | --- | --- | --- | --- | --- |
| **Region** | | **Country** | **Code** | **Stunting prevalence** | **SII** | **CIX** | |
| East and Southern Africa | | Kenya | KEN | -3·19 | -0·20 | -1·09 | |
|  |  | Madagascar | MDG | -6·30 | -2·24 | -0·78 | |
|  |  | Malawi | MWI | -6·79 | 0·80 | -0·89 | |
|  |  | Rwanda | RWA | -4·10 | -9·25 | -4·60 | |
|  |  | Tanzania | TZA | -5·67 | 2·31 | -0·25 | |
|  |  | Uganda | UGA | -7·94 | 1·21 | -0·99 | |
|  |  | Zambia | ZMB | -3·17 | 14·22 | 4·47 | |
|  |  | Zimbabwe | ZWE | 0·27 | 0·59 | -0·19 | |
| West and Central Africa | | Benin | BEN | 10·16 | -9·19 | -1·77 | |
|  |  | Burkina Faso | BFA | -5·88 | -4·20 | -3·39 | |
|  |  | Cameroon | CMR | -3·78 | -11·21 | -7·78 | |
|  |  | Gabon | GAB | -7·53 | 7·00 | -4·39 | |
|  |  | Ghana | GHA | -7·40 | 3·52 | -2·89 | |
|  |  | Mali | MLI | 1·81 | -6·60 | -1·60 | |
| Middle East | | Egypt | EGY | -3·32 | 13·66 | 6·05 | |
|  |  | Jordan | JOR | -1·76 | 2·71 | 3·38 | |
| South and East Asia | | Bangladesh | BGD | -11·81 | -2·07 | -3·31 | |
|  |  | Nepal | NPL | -16·97 | -10·66 | -8·63 | |
|  |  | Cambodia | KHM | -10·53 | -6·74 | -5·00 | |
| Latin America and Caribbean | | Bolivia | BOL | -5·67 | -1·99 | -5·79 | |
|  |  | Brazil | BRA | -6·75 | 32·98 | 36·94 | |
|  |  | Colombia | COL | -4·47 | 6·66 | 2·37 | |
|  |  | Dominican Republic | DOM | -3·33 | 13·86 | 10·52 | |
|  |  | Haiti | HTI | -8·02 | 2·15 | -3·24 | |
|  |  | Peru | PER | -8·72 | 6·08 | -6·60 | |
| **Correlation coefficients** | | | | | | | |
|  |  | |  | **Stunting** | | **SII** | |
|  |  | |  | **Rho** | **P-value** | **Rho** | **P-value** |
|  | **SII** | | | -0·04^†^ | 0·7 |  |  |
|  | **CIX** | | | 0·6^†^ | <0·001 | 0·7^†^ | <0·001 |

^†^Spearman's Rank Correlation Coefficient.

SII: slope index of inequality; CIX: concentration index.
